# Validation and applicability of an alternative method for dialysis water and dialysate quality analysis

**DOI:** 10.1590/2175-8239-JBN-2019-0203

**Published:** 2020-04-30

**Authors:** Gabriela Corrêa Carvalho, Adriana Bugno, Adriana Aparecida Buzzo Almodovar, Fernando Pontes de Lima e Silva, Terezinha de Jesus Andreoli Pinto

**Affiliations:** 1 Universidade de São Paulo, Faculdade de Ciências Farmacêuticas, Departamento de Farmácia, São Paulo, SP, Brasil; 2 Instituto Adolfo Lutz, São Paulo, SP, Brasil

**Keywords:** Renal Dialysis, Water Quality, Validation Studies, Endotoxins, Microbiological Analysis, Diálise Renal, Qualidade da Água, Estudos de Validação, Endotoxinas, Análise Microbiológica

## Abstract

**Introduction:**

In hemodialysis, patients are exposed to a large volume of water, which may lead to fatal risks if not meeting quality standards. This study aimed to validate an alternative method for monitoring microbiological quality of treated water and assess its applicability in dialysis and dialysate analysis, to allow corrective actions in real-time.

**Methods:**

Validation and applicability were analyzed by conventional and alternative methods. For validation, *E. coli* standard endotoxin was diluted with apyrogenic water in five concentrations. For the applicability analysis, treated water for dialysis was collected from different points in the treatment system (reverse osmosis, drainage canalization at the storage tank bottom, reuse, and loop), and dialysate was collected from four machines located in different rooms in the hemodialysis sector.

**Results:**

The validation results were in accordance with the Brazilian Pharmacopoeia acceptance criteria, except for the last two concentrations analyzed. In addition, the ruggedness criterion performed under the US Pharmacopoeia was in agreement with the results.

**Discussion:**

A limiting factor in the applicability analysis was the absence of the endotoxin maximum permitted level in dialysate by the Brazilian legislation. When comparing the analysis time, the alternative method was more time-consuming than the conventional one. This suggests that the alternative method is effective in the case of few analyses, that is, real-time analyses, favoring corrective actions promptly. On the other hand, it does not support the implementation of the alternative method in a laboratory routine due to the high demand for analyses.

## RESUMO

**Introdução:**

Na hemodiálise, os pacientes são expostos a um grande volume de água, o que pode levar a riscos fatais se não cumprir com padrões de qualidade. Este estudo teve como objetivo validar um método alternativo para monitorar a qualidade microbiológica da água tratada e avaliar sua aplicabilidade em análises de diálise e dialisato, para permitir ações corretivas em tempo real.

**Métodos:**

A validação e aplicabilidade foram analisadas por métodos convencionais e alternativos. Para validação, a endotoxina padrão de *E. coli* foi diluída com água apirogênica em cinco concentrações. Para a análise de aplicabilidade, a água tratada para diálise foi coletada em diferentes pontos do sistema de tratamento (osmose reversa, canalização de drenagem no fundo do tanque de armazenamento, reutilização e circuito) e o dialisato foi coletado em quatro máquinas localizadas em diferentes salas do setor de hemodiálise.

**Resultados:**

Os resultados da validação obedeceram aos critérios de aceitação da Farmacopeia Brasileira, com exceção das duas últimas concentrações analisadas. Além disso, o critério de robustez realizado sob a Farmacopeia dos EUA estava de acordo com os resultados.

**Discussão:**

Um fator limitante na análise de aplicabilidade foi a ausência do nível máximo permitido de endotoxina no dialisato pela legislação brasileira. Ao comparar o tempo de análise, o método alternativo consumiu mais tempo que o convencional. Isso sugere que o método alternativo é eficaz no caso de poucas análises, ou seja, análises em tempo real, favorecendo ações corretivas imediatamente. Por outro lado, não suporta a implementação do método alternativo em uma rotina de laboratório devido à alta demanda por análises.

## INTRODUCTION

Nowadays, patients with chronic renal disease can undergo renal replacement therapy, having as options peritoneal dialysis, hemodialysis, or renal transplantation depending on the disease stage and evolution.^1^^,^ 2

Hemodialysis is a widely used procedure, and during the treatment, patients are exposed to a large volume of water. If the water used does not comply with the quality standards required by supervisory bodies, it can represent a risk to patients and may lead to death.^3^^,^ 4

Chemical and microbial contaminants may be harmful to patients on hemodialysis treatment.^5^ Aluminum, chloramine, fluoride, copper, and zinc can be pointed as chemical contaminants, while the most common microorganisms found as contaminants in the water system are *Pseudomonas, Acinetobacter, Flavobacterium, Alcaligenes, Serratia*, all Gram-negative bacteria, and *Mycobacterium.* 1 ^,^ 6

Among components derived from Gram-negative bacteria membranes, endotoxins, which are part of the lipopolysaccharide complex, can be highlighted. The lipopolysaccharide complex has a lipidic portion (lipid A), which confers toxicity during bacteria lysis, death, and during its multiplication.^7^^,^ 8

Endotoxins present in the dialysate cannot be retained by a damaged dialyzer membrane, reaching the patient’s bloodstream and resulting in a pyrogenic reaction by the stimulation of cytokines released by macrophages.^9^^,^ 10

In the United States of America there were six endotoxin contamination outbreaks between 1973 and 1987, affecting 177 patients, but no deaths were recorded.^9^ In a study carried out in 30 dialysis centers in Germany, it was observed that 12.2% of the water samples and 27.5% of the dialysate samples had endotoxin concentration higher than 5 EU/mL.^11^

In Brazil, a study analyzing dialysis water in the city of São Luis/MA, found that 100% and 33.33% of the samples presented endotoxins in the pre- and post-treatment, respectively.^12^

Such incidents indicate that hemodialysis units, besides guaranteeing quality water, must also have a well-established protocol for disinfection, since the bicarbonate concentrate, a component of the dialysis solution, is susceptible to bacterial contamination and elevation of endotoxin levels.^1^

Glucose may be present in the dialysis solution in order to control glycemic levels and optimize the diabetes treatment, but such as bicarbonate, it is a source of carbon and its presence may increase not only bacterial contamination but also endotoxins concentration.^13^^,^ 8

The choice of sensitive and specific analytical methods for water analysis is essential for obtaining quality water and guaranteeing patient safety.^14^ Since 1980, the United States Pharmacopoeia describes the Limulus Amebocyte Lysate Test (LAL) as a method for analyzing endotoxins in pharmaceuticals, largely replacing the use of the pyrogen test in rabbits. Although with distinct comprehensiveness, concepts, and scopes, the bacterial endotoxin assay replaces and overcomes the advantages, in most situations, of the *in vivo* pyrogen test.^7^

The Brazilian Pharmacopoeia recommends two types of endotoxin determination tests: the semi-quantitative gel coagulation test and the quantitative photometric test, which is divided into turbidimetric or chromogenic.^15^

A study carried out by Lemgruber et al.^16^ determined that the inherent error of pipetting interferes with the chromogenic method results, which could compromise reliability. The variability of the analyst’s technique is also a factor that can interfere in the LAL tests.^17^

In an attempt to circumvent the different methodological limitations, alternative methods have been developed to provide a higher level of results quality, greater sensitivity, and agility, allowing corrective actions to be taken earlier.^15^

The alternative method must have attributes such as shorter execution time, faster results, automation, easy execution, miniaturization, low cost, and be in agreement with the analytical parameters.^18^ In this context, it is possible to draw attention to the Portable Test System (PTS^®^), which adopts a portable spectrophotometer developed to simplify sample manipulation and standard preparation for each test, either for standard curve construction or for sample testing. Accurate amounts of LAL reagent, endotoxin, and chromogenic substrates are fixed in a fully pyrogen-free cartridge, eliminating technique variability. As it is a miniaturized system, it can be transported to the sampling point, providing accurate results in real time.^17^

However, the implementation of alternative methods must be preceded by careful validation.^15^ The word validate translates the act of documenting that a given procedure is effective and appropriate to its purpose.^19^ For validation to be considered satisfactory, some steps must be executed with mastery, focusing on the qualification of design, installation, operation, and performance.^7^

In the scope of the present work, intrinsic characteristics of the equipment and attributions of its manufacturer led to the performance qualification.

The performance qualification encompasses the evidence that the alternative method is suitable for routine use in compliance with validation criteria, which are regulated by pharmacopeias and internationally known organizations such as the Parenteral Drug Association (PDA)^7^. The recommendations of the validation criteria to be analyzed depends on the analysis type: qualitative, quantitative, or identification.^15^

If it is proven that the alternative method is equivalent, superior, or non-inferior to the conventional used standard, its substitution might be accepted and stimulated by regulatory agencies of some places such as Australia, Europe, Japan, and the United States.^19^

In this context, the purpose of this study was to validate the PTS^®^ and to evaluate its applicability in the monitoring of treated water for dialysis and dialysate for one month in a dialysis unit.

## MATERIAL AND METHOD

### VALIDATION

In order to validate PTS^®^, the control standard endotoxin (CSE) from *Escherichia coli* was diluted with LAL reagent water at five concentrations (0.0625, 0.125, 0.25, 0.5, and 1.0 EU/mL), which were analyzed by the conventional and alternative methods. For the CSE dilutions preparation the vial was shaken vigorously before use for at least 3 minutes and then the serial dilutions were performed, shaken for at least 30 seconds after each dilution.^15^

In addition, the CSE was prepared at the concentration of 2λ for the positive product control (PPC) and positive LAL reagent water control (PWC).^15^

### CONVENTIONAL METHODOLOGY

The conventional methodology was the gel coagulation method. Firstly, the LAL reagent preparation was carried out, the contents of the vial were reconstituted with LAL reagent water, according to the manufacturer’s instructions, and stored in a freezer until the use.

**Figure 1:**
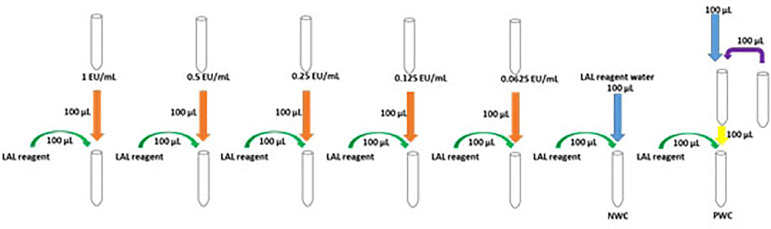
Schematic representation of gel coagulation test.

A volume of 100 µL of LAL reagent was placed in seven apyrogenic test tubes; in five of them, 100 µL of each sample dilution was added, in one tube it was added 100 µL of LAL reagent water to do the LAL reagent negative water control (NWC), and in a seventh tube the PWC was performed, where 100 µL of a solution formed by 100 µL of LAL reagent water plus 100 µL of CSE was added in the concentration of 2λ (Figure 1).^15^

The tubes were then incubated in a water bath for 1 hour at 37±1 ° C, free from vibrations. After this period, the tubes were read and the reaction was considered positive when a firm gel remains intact when the tube is inverted (180º). The reaction was considered negative when there was no gel formation or the clot did not maintain its integrity when the tube was inverted.^15^

### ALTERNATIVE METHOD

The alternative method was the Endosafe PTS^®^ system, with the cartridge that was considered most appropriate, due to the endotoxin limit permitted by legislation for dialysis water samples with a sensitivity of 0.05 to 5 EU/mL.

After the cartridge insertion into the apparatus, a volume of 25 µL of each dilution was pipetted and transferred into each of the four reservoirs. Each reservoir has a channel; in two of them the sample reacts with the LAL reagent and a chromogenic substrate (both present in the cartridge), and in the other two, PPC was performed, because in addition to the LAL reagent, it contains 0.69 EU/mL of endotoxin. The channel optical density is analyzed against a standard curve internally filed.^17^ The analyses were performed by two different analysts, who analyzed the data in triplicate (three cartridges per dilution) totaling six replicates.

The results were analyzed according to the acceptance criteria described in Table 1.

**Table 1:** Parameters description, analysis forms, and acceptance criteria for alternative microbiological and biological methods validation.

Parameters	Analysis Forms	Acceptance Criteria
Accuracy	Recovery percentage determination.	The alternative method recovery should be 100 ± 30% over the conventional one.
Precision	Determination of the coefficient of variation.	The variation coefficient should be less than 30%.
Specificity	Results interpretation	The method should be able to present positive results for the different microorganisms present in the sample.
Limit of detection	Results interpretation, followed by Chi-square test.	At least 50% of the positive results in the conventional method should be positive in the alternative methodology.
Limit of quantification	Results interpretation.	The alternative method should be able to determine the lowest microbial load with accuracy and precision.
Linearity	Calculation of the Square of the correlation coefficient (R^2^) by means of linear regression data analysis.	The alternative method should not have a R^2^ lower than 0.95.
Operational range	Results interpretation.	Determined based on the precision, accuracy, and linearity studies.
Ruggedness	Comparison between replicates of the same analyst and between different analysts.	The alternative method should provide reproducible results even with changes in conditions such as different analysts and different periods.

Source: Adapted from Brazilian pharmacopoeia and United States pharmacopoeia.^15^^,^ 20

### ALTERNATIVE METHOD APPLICABILITY

**Figure 2:**
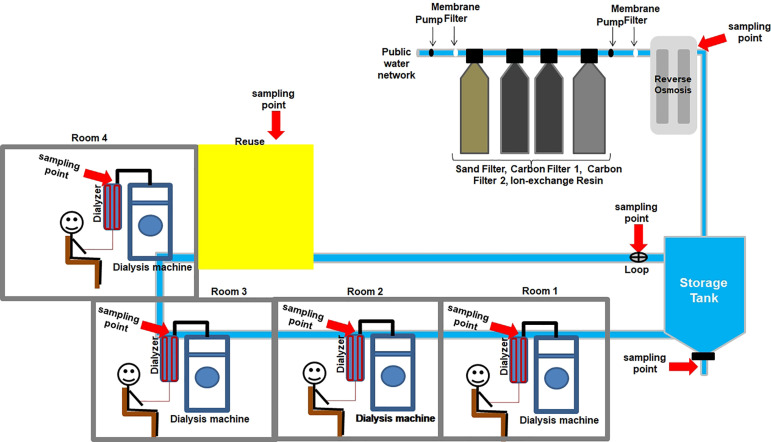
Schematic representation of treated water for dialysis and dialysate sampling.

Samples of treated dialysis water and dialysate were analyzed for bacterial endotoxin presence. Water was collected from four different points in the system: reverse osmosis, drainage canalization at the storage tank bottom, reuse, and loop. The dialysate was collected from four functioning dialysis machines located in different rooms in the hemodialysis sector studied (Figure 2).

In order to monitor this unit for one month, four consecutive collections were carried out, being the first one in the week preceding the chemical disinfection of the water system with 0.2% peracetic acid, which is performed once a month.

A volume of 100 µL of LAL reagent was added to the pyrogenic test tubes and a volume of 100 µL of each sample (reverse osmosis, drainage canalization at the storage tank bottom, reuse, and loop) diluted 1:2 with LAL reagent water was added, according to the maximum valid dilution (MVD) calculation, because the reported sensitivity of the LAL reagent used was 0.125 EU/mL and the endotoxin limit allowed for treated dialysis water is 0.25 EU/mL.^21^

$$\mathrm{MVD}=\frac{\mathrm{endotoxi}n\;\mathrm{limit}}{\lambda }$$

Where λ is the LAL reagent sensitivity expressed on the vial label.^15^

Dialysate samples were diluted at this same concentration. The analyses were performed in triplicate (three cartridges per sample). NWC and PWC were also performed as previously described. In addition, the PPC was performed, where 100 µL of a solution made by 100 µL of the sample plus 100 µL of CSE at the concentration of 2λ was added in tubes containing 100 µL of LAL reagent.

The tubes were then incubated in a water bath at 37±1 °C for 1 hour, avoiding vibrations. After this period the reading was made. Samples that presented positive results for 0.25 EU/mL were submitted to further analysis to verify if endotoxin levels were in the range of 0.25-0.35 EU/mL or greater than 0.5 EU/mL. In parallel, 25 µL of each sample (undiluted) was pipetted into the four reservoirs in each of the PTS^®^ cartridges (triplicate) in order to perform a comparison with the conventional method.

### STATISTICAL ANALYSIS

Minitab^®^ 17 software was used for statistical analysis.

## RESULTS AND DISCUSSION

### VALIDATION

A limitation observed during the analysis was that the replicates of the last concentration, 0.0625 EU/mL, were not detected in any of the replicas because the equipment indicated that it was below the limit of detection; thus, it was not used in the statistical analysis.

### LINEARITY

**Figure 3:**
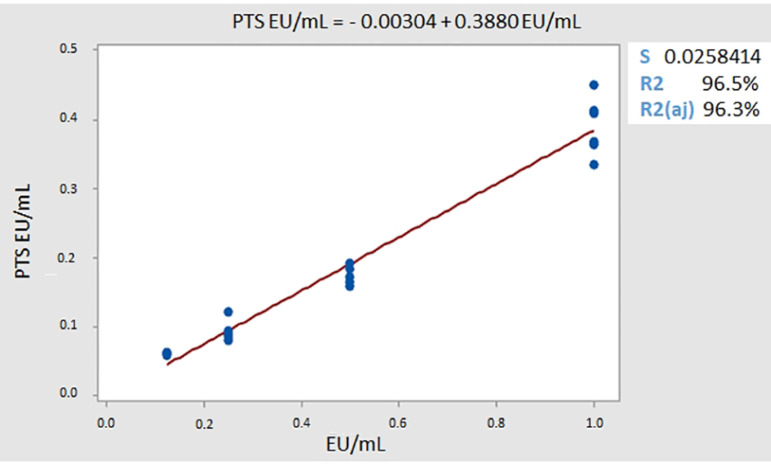
Linear representation of the validation results.

The linear regression analysis (Figure 3) showed a good correlation between the two variables, since the R^2^ was higher than 0.95, suggesting that the results have linearity.^15^ These data corroborate previously performed research where the PTS also presented good linearity.^22^ As the analysis of variance (ANOVA), which measures strength of evidence of the data, was lower than the significance level of 0.05, the linear equation was confirmed.

### ACCURACY

Table 2 shows that the data are not within the recommended range for accuracy, because the recovery percentage should be 100 ± 30%.^15^ When applying the equation obtained by linear regression to correct the data, the values were within the acceptable limit, except for the concentration of 0.125 EU/mL (Table 3). Therefore, concentrations of 1 to 0.25 EU/mL can be considered accurate. In a previous study, PTS^®^ presented significantly different results between the sample concentration and the reading performed by the equipment, which compromised the approval of this criteria.^22^

**Table 2:** Distribution of the conventional method results regarding the alternative method.

Conventional Method (EU/mL)	1	0.5	0.25	0.125
Alternative Method (EU/mL)				
Replicate 1	0.336	0.173	0.089	<0.050
Replicate 2	0.451	0.165	0.121	0.063
Replicate 3	0.365	0.185	0.092	0.061
Replicate 4	0.410	0.158	0.093	<0.050
Replicate 5	0.413	0.193	0.087	<0.050
Replicate 6	0.369	0.193	0.080	0.059
Mean	0.391	0.178	0.094	0.061
Mean Recovery Rate	39.1	35.6	37.6	48.8

Data with "<" were not included in the mean and standard deviation calculations.

**Table 3:** Results obtained from the alternative method validation after correction with the line equation obtained by linear regression.

Conventional Method (EU/mL)	1	0.5	0.25	0.125
Alternative Method (EU/mL)				
Replicate 1	0.874	0.454	0.237	< 0.14
Replicate 2	1.170	0.433	0.320	0.170
Replicate 3	0.949	0.485	0.245	0.165
Replicate 4	1.065	0.415	0.248	< 0.14
Replicate 5	1.072	0.505	0.232	< 0.14
Replicate 6	0.959	0.505	0.214	0.160
Mean	1.015	0.466	0.249	0.165
Mean Recovery Rate	101.5	93.2	99.5	132

Data with "<" were not included in the mean and standard deviation calculations.

### PRECISION

The 1 to 0.125 EU/mL concentrations met the precision criteria, that is, all values were within 30% of the control (Table 4)^15^. Previous studies have also reported that PTS^®^ was accurate.^23^^,^ 24

**Table 4:** Coefficient of variation of the validation data corrected by the line equation.

Endotoxin concentration (EU/mL)	1	0.5	0.25	0.125
Alternative Method (EU/mL)				
Mean	1.015	0.466	0.249	0.165
Standard deviation	0.107	0.038	0.037	0.005
Coefficient of variation (%)	10.55	8.17	14.65	3.12

### SPECIFICITY

For these criteria, only exact concentrations were used. After correcting the data, the cartridge sensitivity limit of 0.050 EU/mL became 0.14 EU/mL. The alternative method under study was characterized by presenting positive results in concentrations greater than its sensitivity (1.0; 0.5; 0.25 EU/mL). These results collectively lead to the confirmation of the method’s specificity (Table 3).^15^

### LIMIT OF DETECTION

The data in Table 3 show that the concentration of 0.125 EU/mL was the lowest that presented 50% of positive results, being, therefore, the detection limit of the alternative method under study. The Brazilian Pharmacopoeia recommends the Chi-square test to evaluate non-inferiority compared to the traditional method, however, due to the small sample size, this test could not be applied.^15^^,^ 25

### LIMIT OF QUANTIFICATION

Based on the results of the precision and accuracy, the limit of quantification of the alternative method under study was 0.25 EU/mL.^15^

### OPERATIONAL RANGE

Based on the results of the precision, accuracy, and linearity, the operational range of the alternative method is from 0.25 to 1.0 EU/mL.^15^

### RUGGEDNESS

**Figure 4:**
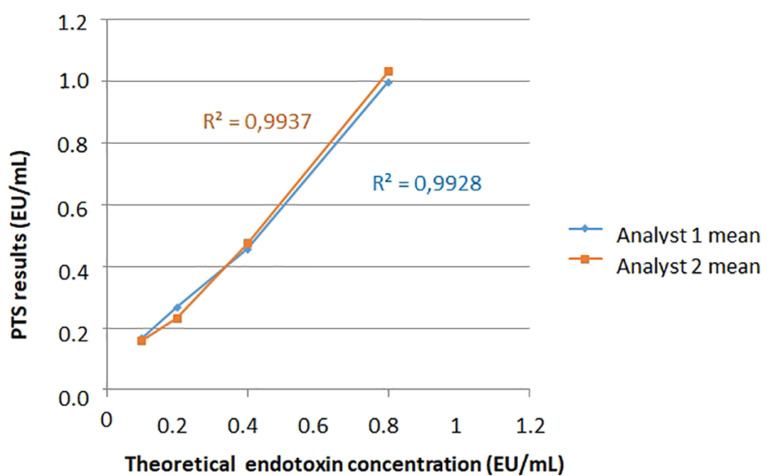
Comparison of PTS^®^ means obtained by the two analysts.

By the proximity of the two analysts’ standard deviations (Table 5) and considering that both coefficient of variations were above 0.95, (Figure 4) it can be said that the alternative method provided reproducible results, confirming its ruggedness.^20^

**Table 5:** Distribution of conventional and alternative method analysis data by day and analysts.

Conventional Method (EU / mL)	Analyst 1				Analyst 2			
Alternative Method (EU / mL)	1	0.5	0.25	0.125	1	0.5	0.25	0.125
Day 1	0.874	0.454	0.237	<0.14	1.065	0.415	0.248	<0.14
Day 2	1.170	0.433	0.320	0.170	1.072	0.505	0.232	<0.14
Day 3	0.949	0.485	0.245	0.165	0.959	0.505	0.214	0.160
Mean	0.998	0.457	0.267	0.168	1.032	0.475	0.231	0.160
Standard deviation	0.154	0.026	0.046	0.004	0.063	0.052	0.017	-

Data with “<” were not included in the mean and standard deviation calculations; “-“calculation were not performed.

The PTS^®^ was validated for samples free of endotoxins or with levels from 0.25 to 1.0. EU/mL, thus it is considered to be effective and suitable for monitoring dialysate and dialysis treated water.^15^^,^ 19

### ALTERNATIVE METHOD APPLICABILITY

Table 6 shows the average results of the analyses performed by the conventional and alternative methods in samples of treated dialysis water. All results, regardless of the method used, are in accordance with the current legislation, i.e., below 0. 25 EU/mL.^21^ These findings are in agreement with a study conducted in Curitiba/PR whose purpose was to evaluate the water quality of six dialysis centers for 12 months, where 85% of the treated dialysis water samples were in accordance with the current legislation.^26^

**Table 6:** Comparison of endotoxin mean levels in dialysate and treated water for dialysis samples by conventional and alternative methods.

Sampling	Method	Endotoxin Level (EU/mL)							
		Dialysis treated water				Dialysate			
		Osmosis	Tank	Reuse	Loop	Mach. 1	Mach. 2	Mach. 3	Mach. 4
1	Conventional	<0.25	<0.25	<0.25	<0.25	< 0.25	< 0.25	< 0.25	< 0.25
	Alternative	<0.14	<0.14	0.140	<0.14	<0.14	<0.14	<0.14	<0.14
2	Conventional	<0.25	<0.25	<0.25	<0.25	< 0.25	0.35> <0.5	< 0.25	< 0.25
	Alternative	<0.14	<0.14	<0.14	<0.14	<0.14	0.17	0.230	<0.14
3	Conventional	<0.25	<0.25	<0.25	<0.25	>0.5	< 0.25	< 0.25	>0.5
	Alternative	<0.14	<0.14	<0.14	<0.14	24.784	<0.14	<0.14	0.974
4	Conventional	<0.25	<0.25	<0.25	<0.25	< 0.25	< 0.25	< 0.25	>0.5
	Alternative	<0.14	<0.14	<0.14	<0.14	<0.14	<0.14	<0.14	1.165

mach. = machine.

When analyzing dialysate samples (Table 6), there was a limitation in determining whether the results are in accordance with the current legislation of the Good Operating Practices Requirements for Dialysis Services, as it only determines that dialysate samples should be collected monthly for heterotrophic plate count, without specifying endotoxin analysis.^21^

Although the Directors Collegiate Resolution (RDC) nº 11 of 2014 does not mention the maximum allowable endotoxin concentration, this study used for analysis purpose the concept of dialysate as being the dilution result, in appropriate proportions, of the polyelectrolytic concentrate for hemodialysis in treated dialysis water.^21^

RDC nº 8 of January 2, 2001 provides for Good Manufacturing Practices for the Polyelectrolytic Hemodialysis Concentrate and determines that the maximum endotoxin concentration allowed is 0.5 EU/mL until the expiration date established by the manufacturer.^27^ Therefore, a prediction for an endotoxin maximum limit of 0.75 EU/mL (0.5 + 0.25 EU/mL) can be assumed.

Disregarding the proportion between water and polyelectrolytic concentrate, the RDC nº 11 of 2014 states that the maximum allowable heterotrophic bacteria for dialysate is 200 CFU/mL. Apparently, this is the sum of the maximum allowable concentration for treated dialysis water, 100 CFU/mL, and for the polyelectrolytic concentrate, 100 CFU/mL, expressed in RDC nº 8 of 2001.^21^^,^ 27

Considering this limit, three samples would be outside the specification limit. However, there were no report of pyrogenic reaction, possibly because the dialysate was collected in the hose that carries water to the polysulfone dialyzer, which can retain endotoxins.^28^ These data corroborate previous research comparing two dialysate membrane types, polysulfone and polyethersulfone. The authors concluded that both are effective in preventing the passage of endotoxins possibly present in the dialysate.^29^

Because of their potential, authors tested membranes made of polysulfone membrane from different brands and observed that there is a permeability difference between them, justifying the performance evaluation by manufacturers.^30^ The dialyzers reprocessing may compromise membrane quality and change its permeability.^9^

A study conducted in 30 dialysis centers in Germany analyzed the endotoxin concentration in treated dialysis water and dialysate samples, which ranged from 0 to 95 EU/mL and 0 to 487 EU/mL, respectively. The authors were concerned about the high levels, proposing a more rigorous regulation. The paper does not, however, refer to dialyzers reuse.^11^

In Brazil, in the city of Ponta Grossa/PR, a survey was conducted to determine the water and dialysate quality of 62 samples collected from the hemodialysis system from November 2003 to April 2004: the presence of endotoxin was observed only in samples preceding reverse osmosis.^31^

In a study performed in Lithuania, treated dialysis water and dialysate samples were analyzed for endotoxin presence; 86% of the treated dialysis water samples and 92% of the dialysate samples had endotoxin levels below 0.25 EU/mL, following the European Pharmacopoeia and the European Best Practice Guidelines for pure dialysis fluid. The study also reports that these percentages could be higher if the monitoring of endotoxin levels was done more frequently.^32^

Klein et al.,^33^ after conducting a study in 51 dialysis centers in the United States, underlined the need for regular endotoxin levels monitoring in the dialysate, which is in agreement with a study conducted in Germany, where the authors call attention to the importance of monitoring, because endotoxin levels in dialysate were higher than in treated dialysis water.^11^

In Brazil, there are dialysis water monitoring programs in some states such as São Paulo and Rio de Janeiro, but they only monitor the treated dialysis water quality.^34^^,^ 35 On the other hand, work carried out in the State of Bahia to propose a monitoring program, also included dialysate monitoring, but only at heterotrophic bacteria level.^36^ Although important, such initiatives fail to address an important aspect of patient safety.

The aforementioned information was obtained using the conventional method and allows defining risk aspects to the patient.^26^ In addition to elaboration and implementation of stricter regulations, the use of methods that provide faster results with greater sensitivity, among other promising characteristics, are recommended.

When comparing the results of the two methods, all samples that were negative in the conventional method were also negative in the alternative one, but one of the four positives samples in the conventional method was negative in the alternative method (Table 7). In parallel, the data in Table 7 were evaluated by Fisher’s Exact Test, showing a significant association between the analyzed methods.

**Table 7:** Comparison of positive and negative means of each method.

	Alternative Method			
		Positive	Negative	Total
**Conventional Method**	Positive	3	1	4
	Negative	0	28	28
	Total	3	29	32

In agreement with the present data, Gee et al.^37^ observed full agreement of negative results in both methods. However, when the results were positive in the conventional method, regardless of the sample dilution, the results obtained in the PTS^®^ were lower, considering the qualitative limit levels (conventional) and those effectively quantified (PTS^®^). The author also reports that both quantitative and qualitative tests should consider a double error margin due to the biological nature of endotoxins present in the sample and the LAL reagent; intrinsic characteristics due to different analysts, laboratories, inputs used, and samples increase the variability and, therefore, it is common for positive samples to diverge in results. The Food and Drug Administration (FDA) and the US Pharmacopoeia admit this limitation and thus provide an acceptable recovery range in kinetic assays of 50 to 200%.^37^

According to the PTS^®^ manufacturer, for a test to be considered valid, the cartridge reaction channel and the PPC channel coefficients of variation where the sample is analyzed must be lower than 25% (spike, with a known amount of endotoxin, 0.69 EU/mL plus sample). Another parameter to consider is the spike recovery percentage, which should be 50 to 200%.^17^ For all tests performed in the present work, the requirements from the PTS^®^ manufacturer were met, as shown in Table 8. Therefore, it can be considered that both validation and applicability data are in agreement with these three parameters.

**Table 8:** Acceptance Criteria by manufacturer and the respective validation and applicability values obtained.

Acceptance Criteria	Validation Data		Applicability Data	
	Highest value	Lower value	Highest value	Lower value
Sample channels coefficient of variation	0.0 %	10.5 %	0.0 %	21.6 %
Positive product control coefficient of variation	0.0 %	14.3 %	0.0 %	18.3 %
Spike Recovery	184 %	96 %	53%	175 %

According to Williams (2001) *apud* Fukumori^23^, the spike recovery within the acceptance criterion indicates that the analysis is not presenting product interference. This suggests that the treated dialysis water and dialysate did not interfere with endotoxin analysis. Hoever, the manufacturers of the gel coagulation method reagent point out that samples with high ionic concentrations should be carefully analyzed, as lipopolysaccharide aggregation may occur causing a false negative^38^. This potential interference was not evident in the present study.

Bambauer et al.^11^ diluted dialysate samples, which had bicarbonate in their composition, in order to avoid interference caused by this component presence in LAL assays. The authors also pointed out that dialysates composed by different types of polyelectrolytic concentrate did not statistically influence the test results.

In a study analyzing saline interference by hemodialysis concentrates on endotoxin determination methods using LAL, the authors concluded that the gel coagulation method can be satisfactorily used for hemodialysis fluid analysis. The study also reports that the compendial chromogenic test, for demanding more time, had its use limited to specific situations, requiring additional dilutions of the sample.^39^ Because PTS^®^ performs analyzes in a reduced time of 15 minutes, the compendial chromogenic method limitation could be avoided in the present study, despite the samples not being diluted.

A research performing PTS^®^ validation considered as acceptable criteria the channel 1 and 3 coefficient of variation, channel 2 and 4 coefficient of variation, and spike recovery percentage, concluding that PTS^®^ can replace the gel coagulation method.^23^ The other validation criteria, described by official compendia and international organizations, were not applied by the author.^15^^,^ 19 ^,^ 20

A similar study that considered the same three criteria, analyzed samples of biopharmaceuticals and water for injections, concluding that both showed negative results when analyzed by PTS^®^ and by the gel coagulation method. The authors emphasized the fact that PTS^®^ is a miniaturized system, so it reduces the sample volume used in the test, and minimizes analyst manipulation and exogenous contamination risk. They also point out that PTS^®^ provided results in 15 min while the gel coagulation method took 1 h.^40^ In this comparison, the authors did not emphasize the sample and reagent preparation time, not even for the sample number and replicates.

In the present study, the preparation time of samples and reagents in the conventional method was around 45 min, while for PTS^®^ it was 15 min. During the 15 min of PTS^®^, the manipulation of the sample and the cartridge were performed, as well as the data insertion in the device, since there was no need for reagent preparation, which justifies the shorter time in this step.

The analysis took 60 min in the conventional method and, on average, 15 min in PTS^®^. It was observed that the higher the level of endotoxin present in the sample, the shorter the analysis time of the device.

During the applicability study, 8 samples were analyzed in triplicate per day, so the total time spent in the conventional method was 115 min while in PTS^®^ it was 60 min per sample, making a total of 480 min to analyze the 8 samples in triplicate. Therefore, regarding the analysis time, PTS^®^ was fast when it comes to *in loco* monitoring, involving the analysis of a few samples. Whereas in a routine laboratory condition with many samples, PTS^®^ can be too time-consuming.

Monitoring the treated water for dialysis and dialysate quality is of fundamental importance for treatment quality and safety. Therefore, the current legislation determines that monthly endotoxin analysis are made on the dialysate, similar as the determination of the dialysate heterotrophic plate count.

However, the official compendia describe routine testing methods that require high analysis time and high manipulation level by the analyst. The present study showed that PTS^®^, as an automated alternative method, is appropriate in relation to analysis time when dealing with *in loco* and real time analysis. However, for laboratories that perform several daily analyzes, it is more time consuming than the conventional method. Given the validation results and the concentrations chosen for the study and according to the sample type analyzed, PTS^®^ was suitable for samples that are expected to be absent of endotoxins or within the operational range of 0.25 to 1 EU/mL.
